# Supplementary material for: Flash heating process for efficient meat preservation
Source: Nat Commun. 2024 May 8;15:3893. doi: 10.1038/s41467-024-47967-1 (PMC11079066; doi:10.1038/s41467-024-47967-1)
Supplement: Supplementary file 1 — Supplementary Information [file 41467_2024_47967_MOESM1_ESM.pdf]

# Supplementary Information: Flash Heating Process for Efficient Meat Preservation

Yimin Mao[1, 2, a], Peihua Ma[3, a], Tangyuan Li[1, a], He Liu[1, a], Xinpeng Zhao[1], Shufeng Liu[1], Xiaoxue Jia[3], Shaik O. Rahaman[3], Xizheng Wang[1], Minhua Zhao[1], Gang Chen[1], Hua Xie[1], Alexandra H. Brozena[1], Bin Zhou[4], Yaguang Luo[4], Rodrigo Tarté[5], Cheng-I Wei[3], Qin Wang[3], Robert M. Briber[1], Liangbing Hu[1, *]

1. Department of Materials Science and Engineering, University of Maryland, College Park, MD, 20742, USA
2. NIST Center for Neutron Research, National Institute of Standards and Technology, Gaithersburg, MD 20899, USA
3. Department of Nutrition and Food Science, University of Maryland, College Park, MD 20742, USA
4. USDA-ARS, Food Quality and Environmental Microbial and Food Safety Laboratories, Beltsville, MD 20705, USA
5. Department of Animal Science, Iowa State University, Ames, IA 50011, USA

Corresponding author: binghu@umd.edu

## Table of Contents

### Supplementary Figures

Supplementary Figure 1. Registering carbon felt light intensity during flashing heating using high speed camera, which is converted to temperature based on previously reported computation protocol

Supplementary Figure 2. Image analysis of the histological micrographs of UFH-treated beef for the quantification of the surface layer thickness

Supplementary Figure 3. Total ion chromatogram (TIC) overview of acrylamide standard and mass spectrum of acrylamide.

Supplementary Figure 4. Microbial growth tests of untreated and UFH-treated beef during storage of 80 h at 4 ℃

Supplementary Figure 5. Water activity of untreated and UFH-treated beef during storage

Supplementary Figure 6. FTIR spectra FH-treated beef (surface and center) and untreated beef

Supplementary Figure 7. Comparison of histological micrographs of the fresh beef and center part of FH-treated beef

Supplementary Figure 8. Storage and loss modulus (G' and G") as a function of strain of untreated beef during storage

Supplementary Figure 9. Storage and loss modulus (G' and G") as a function of strain of UFH-treated beef during storage

Supplementary Table S1. Spectral library search of the liquid chromatography-mass spectroscopy (LC-MS) results of the UFH-treated meat surface

Supplementary Table S2. Spectral library search of the gas chromatography-mass spectroscopy (GC-MS) results of the UFH-treated meat surface

Supplementary Method. High-performance liquid chromatography-mass spectrometry (HPLC-MS) experiment

**Supplementary Method.** Analysis of the temperature and water content change of beef sample during the UFH treatment

**Supplementary Movie S1.** The UFH process recorded using a high-speed camera at a collection rate of 200 fps (frame per second)

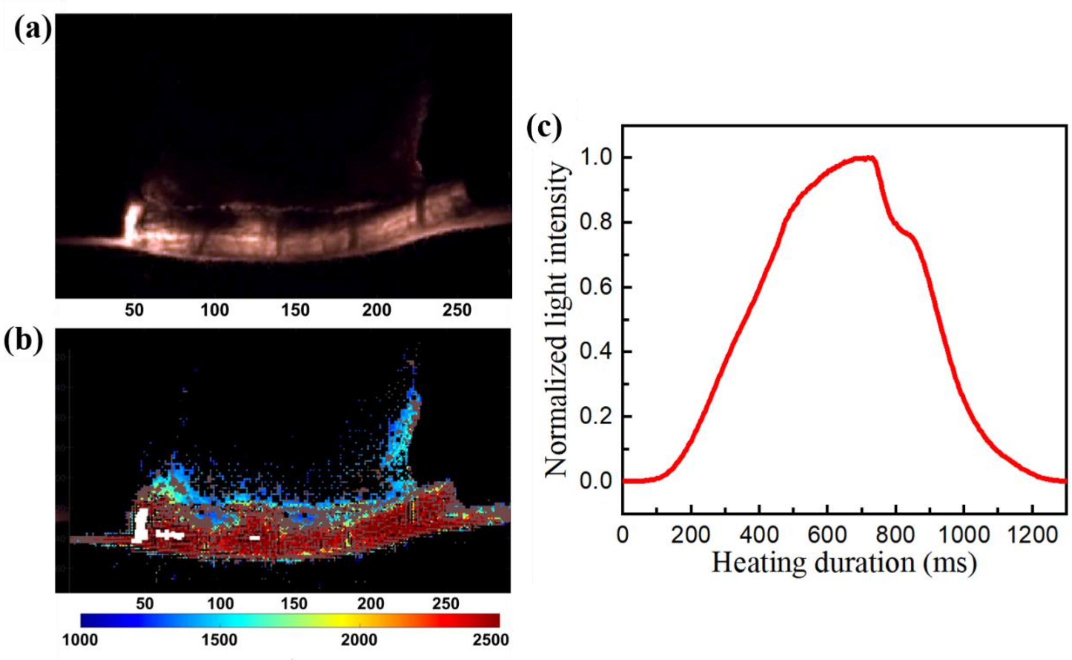


**Figure S1: Registering carbon felt light intensity during flashing heating using high speed camera, which is converted to temperature based on previously reported computation protocol. (a)** A snapshot of the FH treatment at 700 ms. The bright bottom layer is carbon felt. **(b)** Corresponding temperature distribution across the carbon felt. **(c)** Average light intensity of carbon felt during the FH treatment.

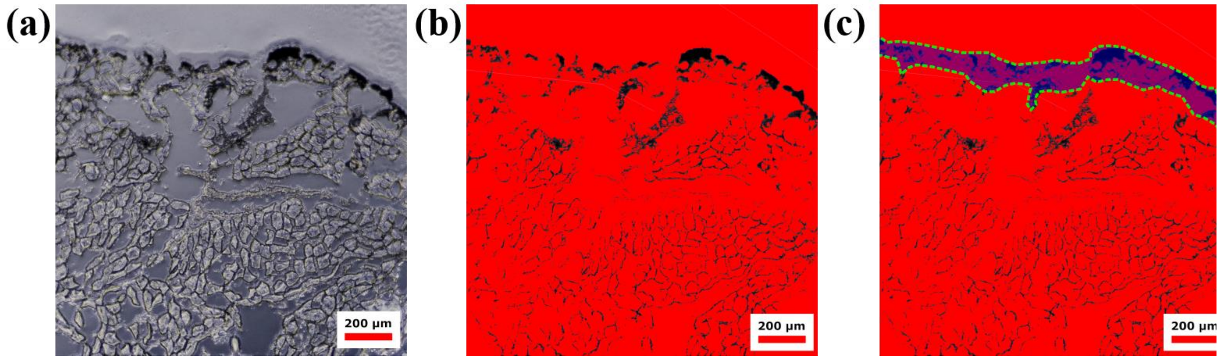


**Figure S2: Image analysis of the histological micrographs of UFH-treated beef for the quantification of the surface layer thickness. (a)** Original histological micrograph. **(b)** Contrast-enhanced image showing a distinct surface layer. **(c)** Defining the surface layer (blue-shaded region); the layer thickness $d = A/2l$, with $A$ being the area of the shaded region and $l$ the contour length of the boundary (dashed green line). Selection of the surface area region was repeated for 5 times and the average value of layer thickness was reported. The analysis was performed using the imageJ software.

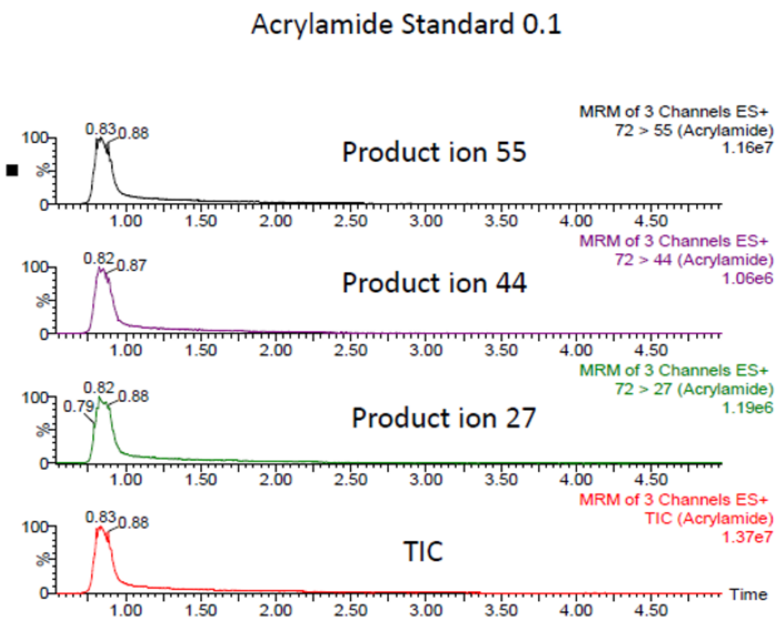


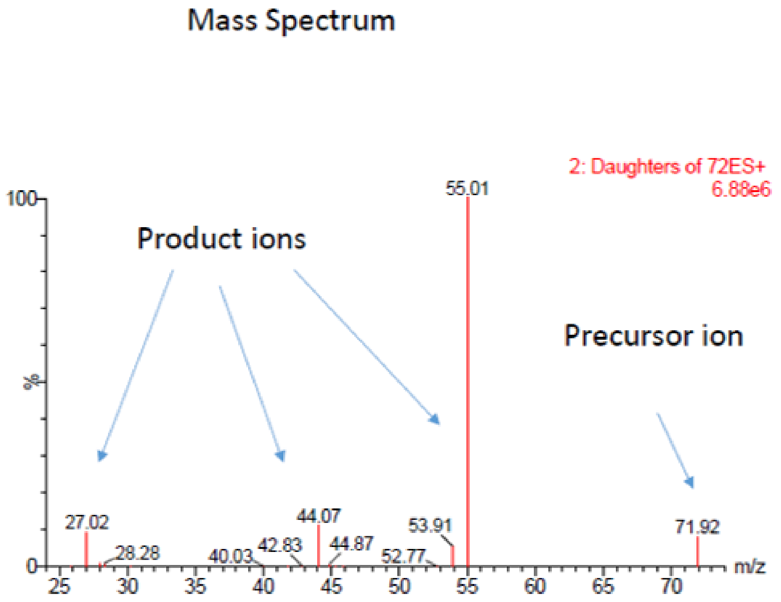


**Figure S3: (a)Total ion chromatogram (TIC) overview of acrylamide standard. (b)mass spectrum of acrylamide.**

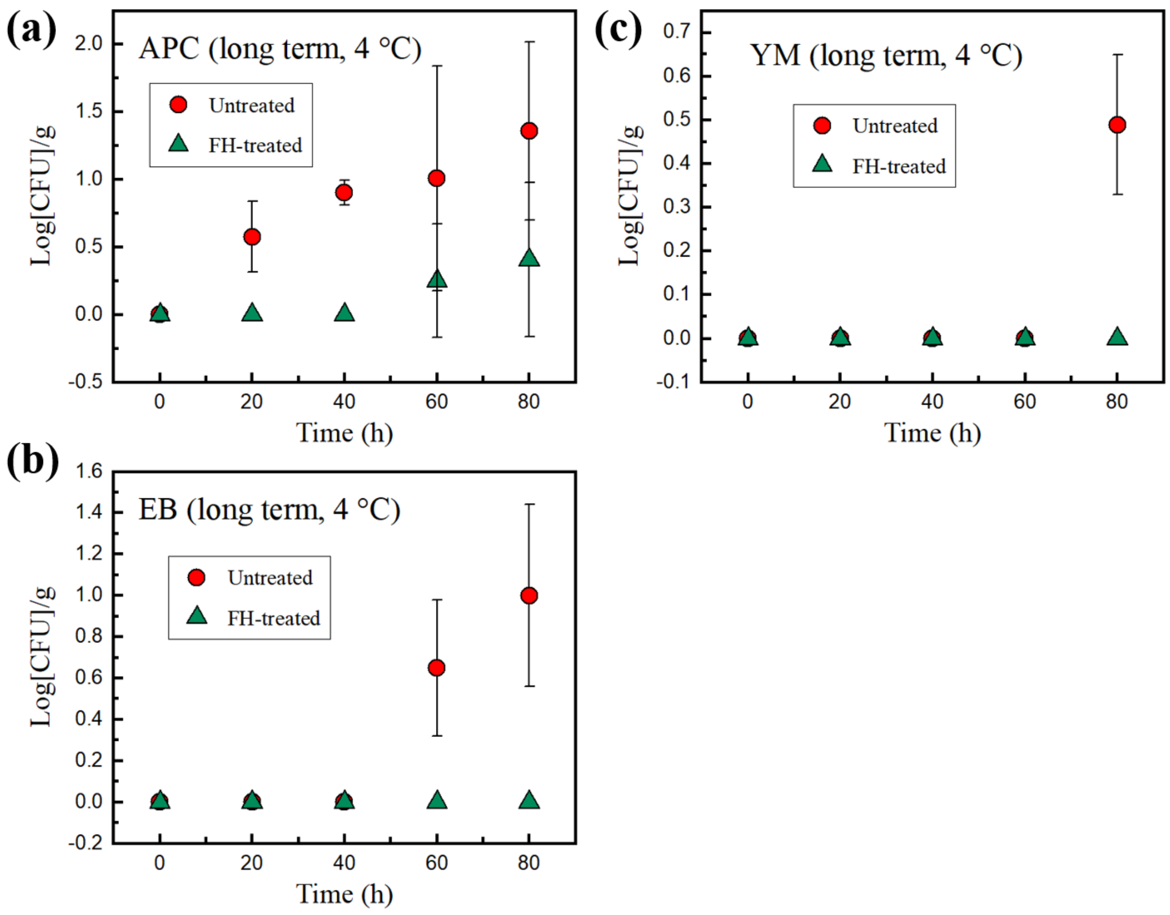


**Figure S4: Microbial growth tests of untreated and UFH-treated beef during storage of 80 h at 4 °C. (a)** Aerobic plate count (APC). **(b)** Enterobacteriaceae (EB). **(c)** Yeast and mold (YM). Error bars indicates one standard deviation of uncertainty.

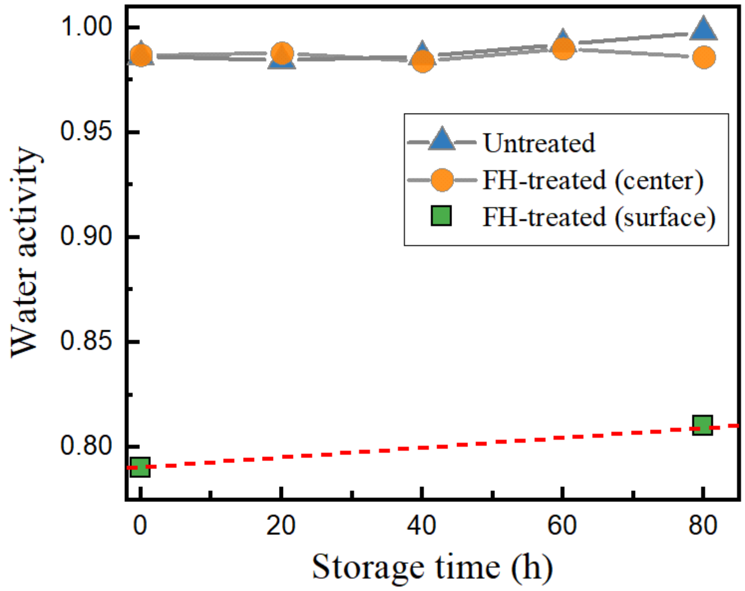


**Figure S5: Water activity of untreated and UFH-treated beef during storage.** Untreated beef, as well as the center part of the UFH-treated beef, maintain a high level of water activity

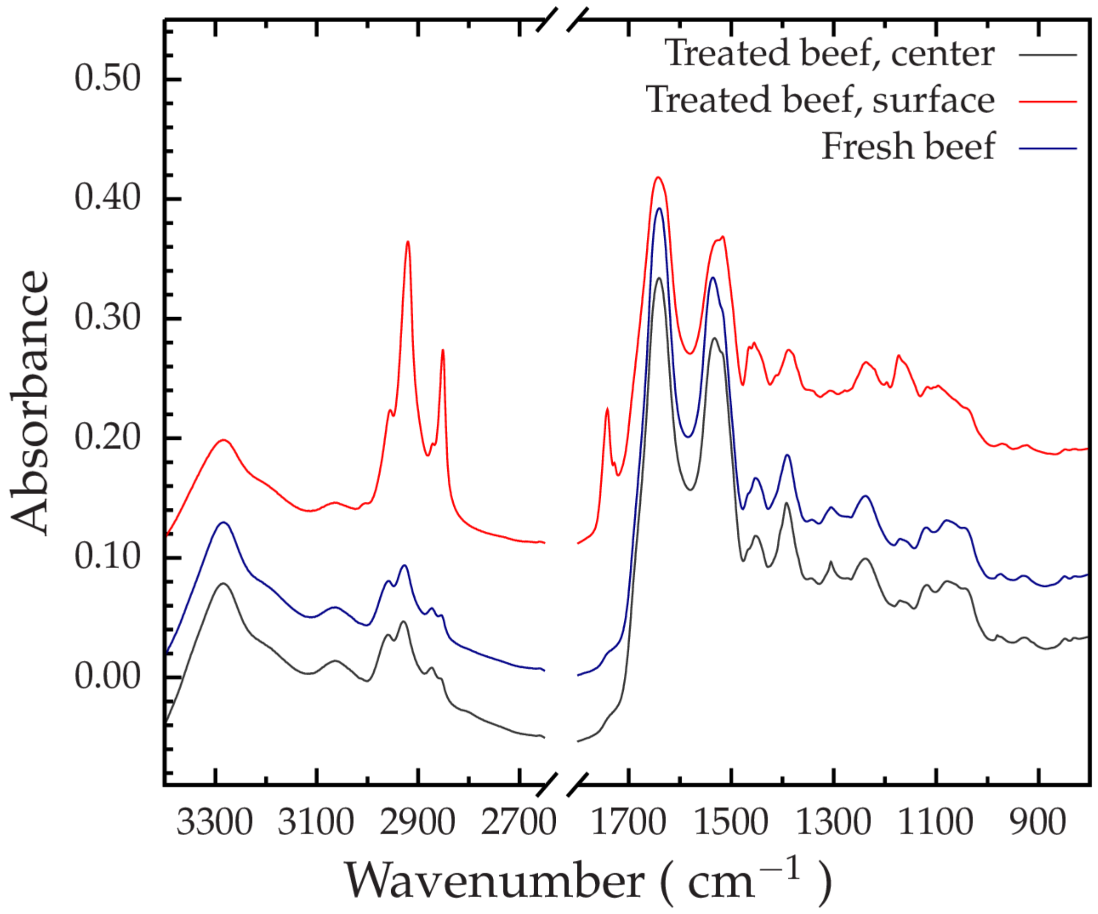


**Figure S6: FTIR spectra FH-treated beef (surface and center) and untreated beef.**

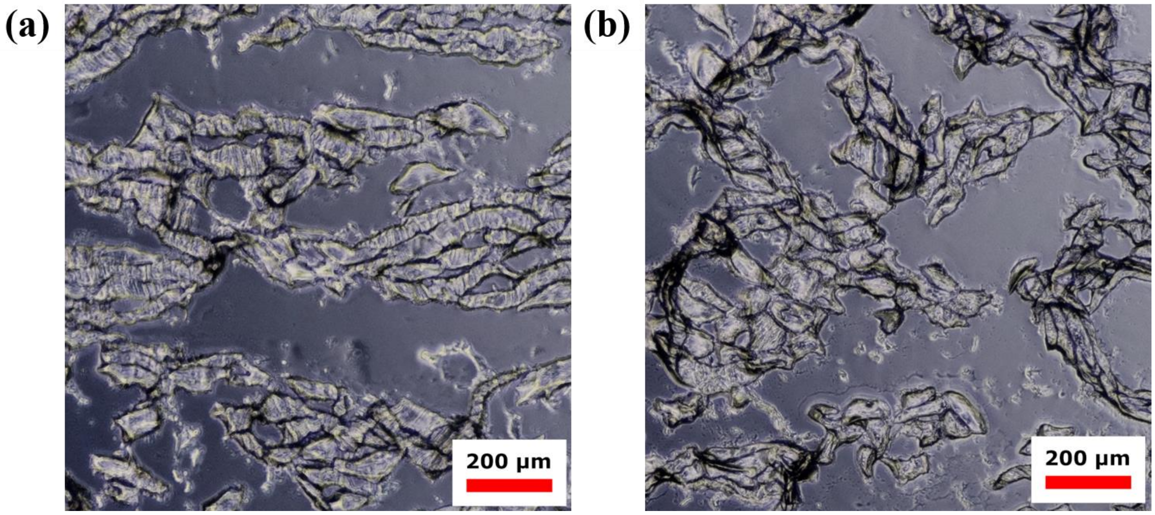


**Figure S7: Comparison of histological micrographs of the (a) fresh beef and (b) center part of the UFH-treated beef.**

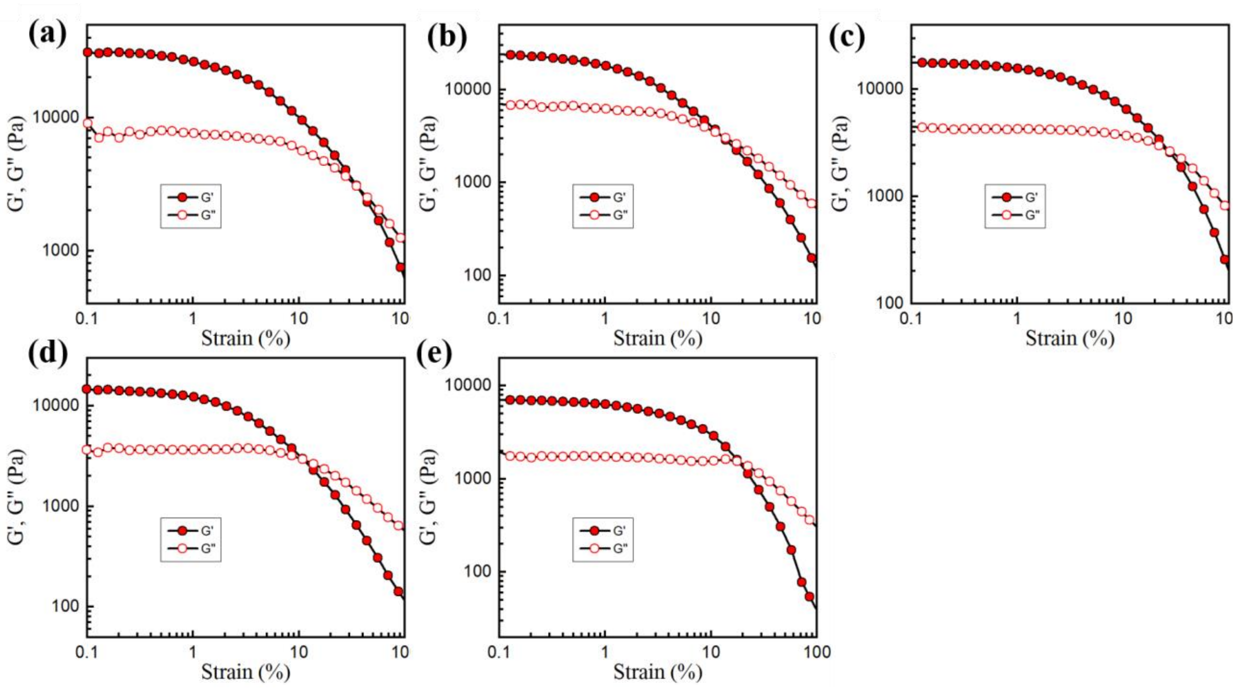


**Figure S8: Storage and loss modulus (G’ and G”) as a function of strain of untreated beef during storage. (a-e)** Rheological data at 20, 40, 60, 80, and 100 h.

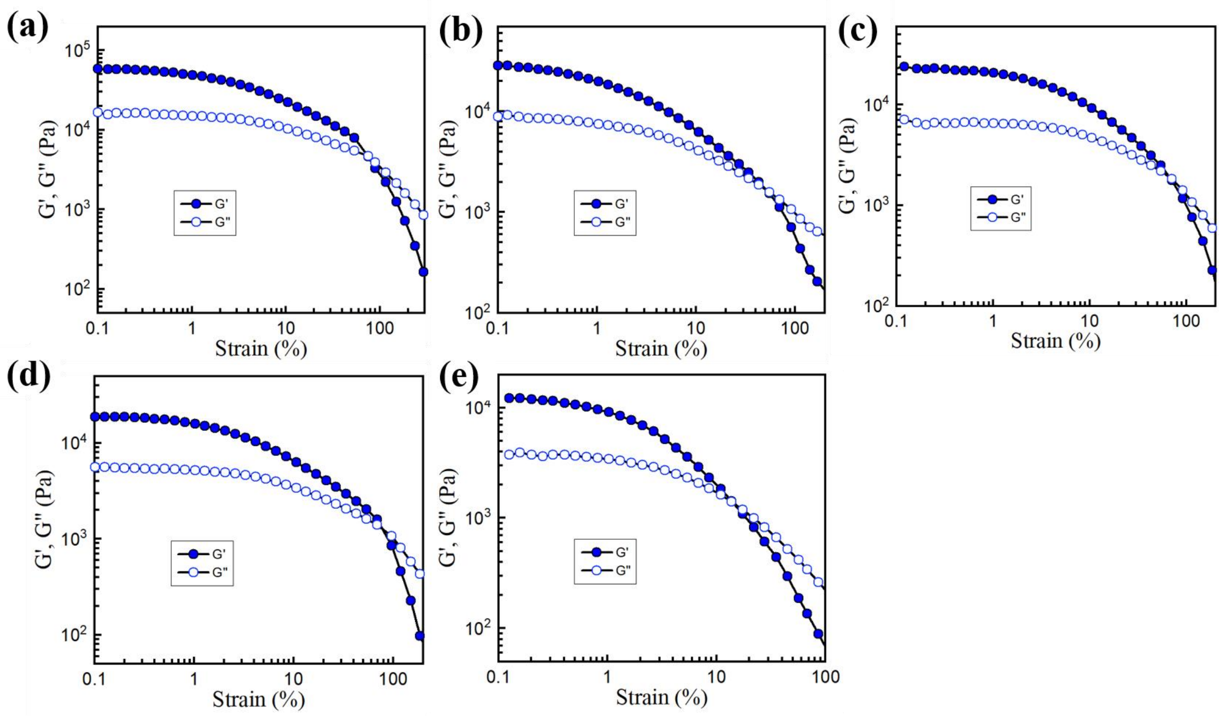


**Figure S9: Storage and loss modulus (G' and G") as a function of strain of UFH-treated beef during storage. (a-e)** Rheological data at 20, 40, 60, 80, and 100 h.

**Table S1.** Spectral library search of the LC-MS results of the UFH-treated meat surface

| Id | Area | ~Retention time (RT) | Compound name | Matches cosine score |
|---|---|---|---|---|
| 253 | 5.45E+07 | 0.3865 | ACETYL-CARNITINE | 0.999 |
| 587 | 5.27E+07 | 0.5829 | ACETYL-CARNITINE | 0.999 |
| 876 | 4.52E+06 | 1.2221 | PROPIONYLCARNITINE | 0.998 |
| 170 | 5.70E+07 | 0.375 | CARNITINE | 0.993 |
| 7394 | 6.15E+05 | 6.7092 | Massbank:LU123902 N-Phenyl-1-naphthylamine\|N-phenylnaphthalen-1-amine | 0.992 |
| 7326 | 1.80E+06 | 6.6092 | palmitoyl carnitine CollisionEnergy:205060 | 0.99 |
| 1156 | 5.89E+07 | 1.659 | DL-PHENYLALANINE CollisionEnergy:102040 | 0.989 |
| 763 | 1.11E+07 | 0.7968 | TYROSINE | 0.989 |
| 407 | 4.86E+07 | 0.421 | CARNOSINE | 0.986 |
| 604 | 5.81E+06 | 0.5945 | Methionine | 0.985 |
| 956 | 3.18E+07 | 1.5061 | INOSINE | 0.984 |
| 626 | 2.62E+07 | 0.6293 | HYPOXANTHINE | 0.983 |
| 5315 | 4.29E+06 | 2.942 | Massbank:RP025502 Hexanoyl-L-Carnitine\|L-Hexanoylcarnitine\|(3R)-3- | 0.982 |

| | | | | |
|---|---|---|---|---|
| | | | hexanoyloxy-4-(trimethylazaniumyl)butanoate | |
| 7611 | 5.87E+06 | 7.1046 | PC(0:0/18:1); [M+H]+ C26H53N1O7P1 | 0.982 |
| 7500 | 1.44E+07 | 6.9234 | PC(0:0/16:0); [M+H]+ C24H51N1O7P1 | 0.981 |
| 7342 | 9.71E+06 | 6.6208 | PC(18:2/0:0); [M+H]+ C26H51N1O7P1 | 0.98 |
| 7262 | 9.48E+06 | 6.4957 | PC(18:2/0:0); [M+H]+ C26H51N1O7P1 | 0.979 |
| 7680 | 1.17E+06 | 7.2036 | PC(0:0/18:1); [M+H]+ C26H53N1O7P1 | 0.978 |
| 7239 | 1.52E+06 | 6.472 | PE(18:2/0:0); [M+H]+ C23H45N1O7P1 | 0.978 |
| 706 | 3.07E+06 | 0.7125 | xanthine CollisionEnergy:102040 | 0.975 |
| 7860 | 2.17E+06 | 7.6943 | PC(0:0/18:0); [M+H]+ C26H55N1O7P1 | 0.974 |
| 7482 | 1.25E+06 | 6.8995 | PC(20:3/0:0); [M+H]+ C28H53N1O7P1 | 0.972 |
| 7349 | 2.68E+06 | 6.6208 | PC(0:0/20:4); [M+H]+ C28H51N1O7P1 | 0.97 |
| 7568 | 3.73E+05 | 7.0395 | PE(22:4/0:0); [M+H]+ C27H49N1O7P1 | 0.97 |
| 5699 | 2.15E+05 | 3.1514 | MassbankEU:SM817801 Cinnamamide\|(E)-3-phenylprop-2-enamide | 0.969 |
| 7586 | 1.17E+06 | 7.0808 | PE(18:1/0:0); [M+H]+ C23H47N1O7P1 | 0.969 |
| 7533 | 5.12E+06 | 6.9588 | PC(0:0/18:1); [M+H]+ C26H53N1O7P1 | 0.968 |
| 949 | 6.87E+06 | 1.5061 | HYPOXANTHINE | 0.964 |
| 7314 | 1.90E+06 | 6.5975 | PE(18:2/0:0); [M+H]+ C23H45N1O7P1 | 0.96 |
| 802 | 3.10E+06 | 0.9104 | 3-hydroxybutyrylcarnitine | 0.957 |
| 7176 | 1.66E+05 | 6.2373 | PC(16:1/0:0); [M+H]+ C24H49N1O7P1 | 0.95 |
| 7184 | 4.62E+05 | 6.2515 | PC(20:5/0:0); [M+H]+ C28H49N1O7P1 | 0.949 |
| 8478 | 2.05E+05 | 9.1848 | cholesta-5,7-dien-3beta-ol | 0.948 |

| | | | | |
|---|---|---|---|---|
| 26 | 7.96E+05 | 0.3172 | HISTIDINE | 0.944 |
| 7656 | 2.86E+05 | 7.1762 | PC(22:4/0:0); [M+H]+ C30H55N1O7P1 | 0.942 |
| 6368 | 3.46E+05 | 3.9168 | OCTANOYLCARNITINE | 0.938 |
| 649 | 4.51E+06 | 0.6293 | INOSINE 5'-MONOPHOSPHATE | 0.926 |
| 6979 | 1.67E+05 | 5.3986 | LAUROYLCARNITINE | 0.906 |
| 8046 | 8.46E+05 | 8.1381 | 16-Hydroxyhexadecanoic acid CollisionEnergy:205060 | 0.897 |
| 7841 | 9.87E+05 | 7.6665 | PE(18:0/0:0); [M+H]+ C23H49N1O7P1 | 0.88 |
| 6759 | 1.12E+05 | 4.7271 | DECANOYLCARNITINE | 0.866 |
| 7472 | 7.26E+05 | 6.8726 | PE(20:3/0:0); [M+H]+ C25H47N1O7P1 | 0.838 |
| 853 | 1.25E+06 | 1.0939 | Spectral Match to S-(5'-Adenosyl)-L-homocysteine from NIST14 | 0.824 |
| 846 | 1.45E+06 | 1.0812 | Spectral Match to Glutathione, oxidized from NIST14 | 0.823 |

**Table S2.** Spectral library search of the GC-MS results of the UFH-treated meat surface

| Compound match | ~RT | Match |
|---|---|---|
| Glycerin | 7.4 | 82.2P |
| 4H-Pyran-4-one,2,3-dihydro-3,5-dihydroxy-6-methyl- | 7.96 | 92.2P |
| Octanoic acid | 8.03 | 72.5P |
| 2(3H)-Furanone,dihydro-4-hydroxy- | 8.14 | 95.9P |
| Decanoic acid | 9.14 | 74.7P |
| Dodecanoic acid | 10.19 | 75.3P |

**Supplementary Method.** High-performance liquid chromatography-mass spectrometry (HPLC-MS) experiment

In our HPLC-MS experiment, we utilized two distinct setups to analyze complex samples. The first setup employed an Orbitrap Q-Exactive mass spectrometer, using a Waters ACQUITY BEH C18 column with a fine particle size of 1.7 µm and a length of 50 mm. This setup was designed for high-resolution and accurate mass measurements, suitable for the analysis of a wide range of analytes in complex mixtures. We used a gradient of water containing 0.1% formic acid (Solvent A) and acetonitrile containing 0.1% formic acid (Solvent B), which was increased from 0% to 95% B over 10 minutes. The second setup involved the Waters Xevo TQ-XS, a triple quadrupole mass spectrometer, which excels in quantitative analysis. This setup used a faster gradient over 5 minutes and an Imtakt Intrada Amino Acid column, specialized for amino acid analysis. Both methods showcase the versatility of HPLC-MS in handling different analytical requirements, from broad-spectrum analysis with the Orbitrap to targeted, sensitive detection with the triple quadrupole.

**Supplementary Method.** Analysis of the temperature and water content change of the beef sample during the UFH treatment.

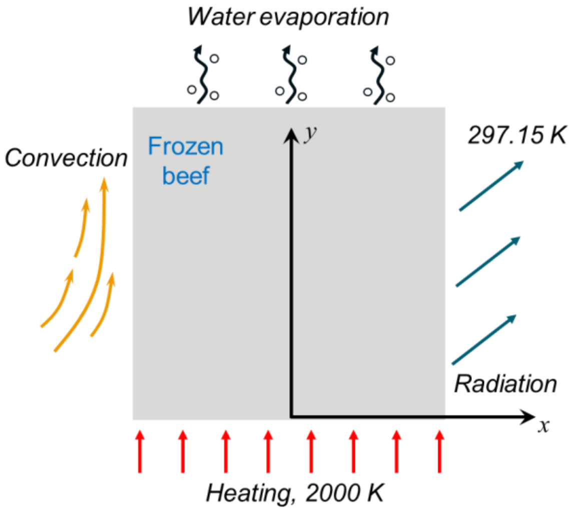


**Figure S10: Schematic diagram of heat and water vapor transport during ultra-high temperature exposure of frozen beef at -20 °C.**

At the initial time ($t = 0$), a carbon heater, powered by a direct current (DC) supply and located at the bottom of the frozen beef, generates a high temperature of 2000 K lasting for 1 second. This heat is transferred to the frozen beef, potentially increasing its temperature, melting the ice, and causing the water within the beef to evaporate. Concurrently, the beef exchanges heat with its surroundings mainly through convection and radiation. By assuming uniformity in the beef, the variation of the temperature ($T(t,x,y)$) and water content ($C(t,x,y)$) within it can be approximately described as follows:

$$\rho C_p \frac{\partial T(t,x,y)}{\partial t} = k\nabla^2 T(t,x,y) \tag{1}$$

$$\frac{\partial C(t,x,y)}{\partial t} = D\nabla^2 C(t,x,y) \tag{2}$$

where $\rho$ (kg/m$^3$) is the density of the beef, $C_p$ (J/(kg·K)) denotes the heat capacity of the beef, $k$ indicates the temperature-dependent thermal conductivity**Error! Reference source not found.**, and $D$ represents the diffusion coefficient of the water**Error! Reference source not found.**. Here we assume that water mass transfer in the beef does not initiate until the beef is unfrozen, meaning ice sublimation and mass transfer can be neglected before the beef melts ($D = 0 \text{ m}^2/\text{s}$). We employed the apparent heat capacity method to solve the heat transfer equation (Eq. (1)), assuming that beef melting occurs within a specific temperature interval. The latent heat of melting is taken into account by the modification of the specific heat capacity, which is expressed by:

$$c_p = \theta_s c_{p,s} + \theta_l c_{p,l} + L_{s\to l}\frac{\partial \alpha_m}{\partial T} \tag{3}$$

where $\theta_s$ and $c_{p,s}$ represent the volume fraction and specific heat capacity of the beef before melting, respectively; $\theta_l$, and $c_{p,l}$ represent the volume fraction and specific heat capacity of the beef before melting. $L_{s\to l}$ is the latent heat of melting, and $\alpha_m$ is defined as the mass fraction of the melted beef. Assuming that water evaporation occurs only on the surface of the beef, the boundary conditions of the heating surface and other surfaces for heat transfer equation (Eq. (1)), including convective, radiative, and evaporative heat transfer, can be described as follows, respectively:

$$k\nabla T = h(T_{air} - T) + \varepsilon\sigma(T_h^4 - T^4) + DL_{l\to g}\nabla C \tag{4}$$

where $h$ (W/(m$^2$·K)) is the convection heat transfer coefficient, $T_{air}$ (K) and $T_h$ (K) denote the surrounding air temperature and the heater temperature, respectively, $L_{l\to g}$ is defined as the molar latent heat of vaporization, $\varepsilon$ refers to the surface emissivity of the beef, σ is the Stefan-Boltzmann

constant, and $DL_{l\rightarrow g}\nabla C$ denotes the heat flux out due to moisture vaporization. According to the conservation of mass, the boundary condition for mass transfer equation (Eq. (2)) is as follows:

$$D\nabla C = k_c(C_b - C) \quad (5)$$

where $C_b$ denotes the air moisture concentration and $k_c = h_m/(\rho_{p,l} \cdot C_m)$ is defined as the mass transfer coefficient of the water. In this context, $h_m$ is mass transfer coefficient in mass units and $C_m$ is the specific moisture capacity. In the simulations, the initial temperature ($T_0$) is set at 253.15 K, and the initial water concentrations of the beef is roughly estimated by $C_0 = \varphi\rho_{p,l}/M_{H_2O}$, where $\varphi = 0.75$ represents the water content. The surrounding air temperature is 293.15 K and the convection heat transfer coefficient is 13.75 W/(m²·K). More details can be found in Table 1.

**Table S1.** List of the parameters used for simulation.

| Parameters | Value | Description |
|---|---|---|
| $T_{air}$ | 293.15 K | surrounding air temperature |
| $T_0$ | 253.15 K | initial temperature of the beef |
| $h$ | 13.75 W/(m²·K) | convection heat transfer coefficient |
| $M_{H_2O}$ | 0.018 kg/mol | water molecular weight |
| $C_0$ | 43542 mol/m³ | initial moisture concentration |
| $C_b$ | 1161.1 mol/m³ | air moisture concentration |
| $C_m$ | 0.003 | specific moisture capacity |
| $h_m$ | $1.67\times10^{-6}$ kg/(m²·s) | mass transfer coefficient in mass units |
| $k_c$ | $5.33\times10^{-7}$ m/s | mass transfer coefficient |
| $L_{l\rightarrow g}$ | 41400 J/mol | molar latent heat of evaporation |
| $L_{s\rightarrow l}$ | 180.8 kJ/kg | latent heat of melting |
| $\rho_{p,l}$ | 1045 kg/m³ | density of melted beef |
| $\rho_{p,s}$ | 961 kg/m³ | density of frozen beef |
| $c_{p,l}$ | 3510 J/(kg·K) | specific heat capacity of melted beef |
| $c_{p,s}$ | 2090 J/(kg·K) | specific heat capacity of frozen beef |

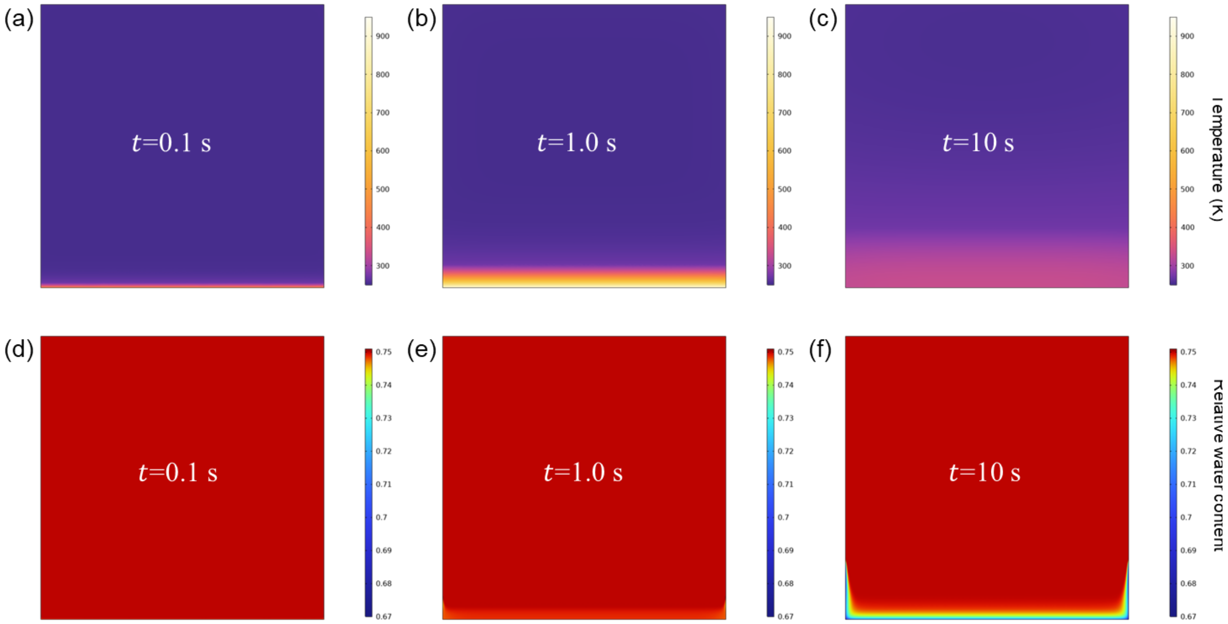


**Figure S11: Simulated (a-c) temperature distribution and (d-f) relative water content distribution in the heated beef.**

## References

1. Willix, J., Lovatt, S. J, Amos, N. D. Additional thermal conductivity values of foods measured by a guarded hot plate. *J. Food Eng.*, 1998, **37(2)**, 159-174.

2. Trujillo, F. J., Wiangkaew C., Pham Q. T. Drying modeling and water diffusivity in beef meat. J. Food Eng., 2007, **78(1)**, 74-85.
