# Supplementary material for: Flash heating process for efficient meat preservation
Source: Nat Commun. 2024 May 8;15:3893. doi: 10.1038/s41467-024-47967-1 (PMC11079066; doi:10.1038/s41467-024-47967-1)
Supplement: Supplementary file 3 — Description of Additional Supplementary Files [file 41467_2024_47967_MOESM3_ESM.pdf]

Description of Additional Supplementary Files

File Name: Supplementary Movie S1
Description: Ultra-high temperature flashing heating process.
